# Effects of Dalcroze Eurhythmics Exercise Versus Multicomponent Exercise on Physical and Cognitive Function, and Falls in Older Adults: The EPHYCOS Randomized Controlled Trial

**DOI:** 10.1002/adbi.202400089

**Published:** 2024-05-03

**Authors:** Mélany Hars, Natalia Fernandez, François Herrmann, René Rizzoli, Serge Ferrari, Christophe Graf, Patrik Vuilleumier, Andrea Trombetti

**Affiliations:** ^1^ Division of Bone Diseases Department of Medicine Geneva University Hospitals and Faculty of Medicine Geneva 1205 Switzerland; ^2^ Division of Geriatrics and Rehabilitation Department of Rehabilitation and Geriatrics Geneva University Hospitals and Faculty of Medicine Geneva 1226 Switzerland; ^3^ Laboratory for Behavioral Neurology and Imaging of Cognition Department of Neuroscience Faculty of Medicine University of Geneva Geneva 1205 Switzerland

**Keywords:** aging, cognitive function, exercise, falls, physical function

## Abstract

Currently, robust evidence is lacking to support one exercise type over another in the prevention of physical and cognitive decline and falls among older adults, primarily because of the lack of comparative trials of proven interventions. Therefore, a 12‐month randomized, single‐blind, comparative effectiveness trial is conducted, in which 142 older adults at high risk for falls are randomized (1:1) to receive an evidence‐based Dalcroze Eurhythmics (DE) exercise program (once weekly, group‐based) or an evidence‐based multicomponent (MULTI) exercise program incorporating balance, functional, and strength training activities (twice weekly, group‐ and home‐based), for 12 months. The primary outcome is gait variability under dual‐task at 12 months. At 12 months, the DE group has significant improvements compared with MULTI group on gait under both dual‐task (adjusted β for stride variability: −2.3, 95%CI, −3.1 to −1.4; *p* < 0.001) and single‐task, and on a variety of secondary physical and cognitive/executive function outcomes. The adjusted hazard ratio for falls is 0.58 (95%CI, 0.37 to 0.93) for the DE group compared with MULTI group. In conclusion, DE exercise is more effective than MULTI exercise in improving physical and cognitive function and reducing falls in older adults. The mechanisms underlying DE exercise‐induced benefits remain to be fully elucidated.

## Graphical abstract

In this randomized clinical trial conducted among 142 community‐dwelling older adults at high risk for falls over 12 months, a Dalcroze Eurhythmics exercise intervention (once weekly, group‐based) improved a variety of physical and cognitive/executive function outcomes compared with a multicomponent exercise intervention (twice weekly, group‐ and home‐based), and reduced falls.

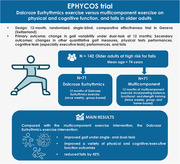

## 1 Introduction

Aging is accompanied by a myriad of health concerns, among which falls are one of the most common and devastating. Falls affect over one in three adults aged above 65 years living in the community each year, and remain a leading contributor to excess disability, nursing home placement, morbidity and mortality.^[^ ^1^, ^2^, ^3^, ^4^ ^]^ With the number of older persons projected to more than double by 2050, the burden of falls is on track to reach epidemic proportions. To identify and implement effective fall prevention interventions is timely and worthwhile.

Interventional studies have identified exercise as one of the most effective for counteracting key risk factors for falls, including physical impairments, and reducing falls in older adults. Several recent systematic reviews and meta‐analyses support the protective effect of various exercise types on falls in older adults.^[^ ^2^, ^5^, ^6^, ^7^, ^8^, ^9^, ^10^, ^11^, ^12^, ^13^ ^]^ Thus, all current clinical practice guidelines for fall prevention and management strongly recommend exercise, especially interventions incorporating balance training as a core component.^[^ ^6^, ^12^, ^14^ ^]^ To date, multicomponent exercise interventions (most commonly involving balance, functional, and strength training) have been the most widely disseminated. Despite important strides made in the field of exercise, some key gaps remain in the evidence base. Especially, robust evidence is lacking to date to support one exercise intervention type over another for the prevention/management of physical decline and falls among community‐dwelling older adults, primarily because of the lack of head‐to‐head comparative effectiveness trials of proven interventions. Filling this important gap is crucial to improve clinical and health policy decision making.

In the recent decade, physical‐cognitive exercise interventions, especially of multitasking nature, have emerged as promising strategies for falls reduction in the older population by providing potential enhanced value through their beneficial effects on key cognitive processes. However, evidence that combination of physical and cognitive training is more effective in fall prevention compared to multicomponent physical training remain inconclusive.^[^ ^15^ ^]^

Dalcroze Eurhythmics (DE) (i.e., a music‐based multitask exercise intervention) has previously been shown to improve dual‐task abilities (i.e., walking while performing a cognitive task), improve executive functioning and reduce falls, in a passive‐controlled trial.^[^ ^16^, ^17^, ^18^, ^19^ ^]^ Notably, the DE intervention, which provides a greater commitment to multiple‐task training than other exercise forms, reduced gait variability under dual‐task condition. Dual‐task/multi‐tasking are an essential part of older adults' everyday life, while gait variability measures, especially under dual‐task, have been strongly linked to fall risk.^[^ ^20^, ^21^, ^22^, ^23^, ^24^, ^25^ ^]^

Improved executive functioning, and associated functional plasticity, also has the potential to be an important yet largely unexplored mechanism by which exercise may improve physical function and reduce falls.^[^ ^25^, ^26^, ^27^, ^28^, ^29^, ^30^, ^31^ ^]^ Executive functions encompass the set of higher‐order cognitive processes required for goal‐directed, adaptive and flexible behavior in novel, demanding, changing or complex everyday life situations. Deficits in executive functions may increase the propensity to fall via various pathways including impaired gait and balance.^[^ ^25^, ^26^, ^27^, ^29^, ^32^ ^]^

Based on our previous passive‐controlled trial, we designed the EPHYCOS trial with the aim to determine the effectiveness of an evidence‐based DE exercise intervention, compared with an evidence‐based multicomponent (MULTI) exercise intervention (incorporating balance, functional, and strength training), in improving physical and executive function and reducing falls.

## 2 Measurements

### 2.1 Outcomes

#### 2.1.1 Gait Outcomes

Gait was assessed under single‐ and dual‐task using an electronic pressure sensitive walkway (GAITRite; CIR Systems Inc, Havertown, Pennsylvania), as described previously.^[^ ^18^ ^]^ Briefly, the participants were asked to walk under different conditions including at a self‐selected usual speed as a single task, and a self‐selected speed while simultaneously count aloud backward by 1 starting from 50, as a dual task, without specific instruction regarding task prioritization. Gait variability was measured using the coefficient of variation (CV). As previously reported, under the dual‐task condition, intraclass correlation coefficients (2,1) for gait variability measures in this population are above 0.68. The standard error of measurement value is 0.99% for CV of stride length.

#### 2.1.2 Physical Outcomes

The following physical performances tests were administered: the Short Physical Performance Battery (SPPB), a composite of three timed tests (i.e., standing balance test, 4‐m gait speed test, and five‐repetition chair stand test),^[^ ^33^ ^]^ the Timed Up & Go test (TUG),^[^ ^34^ ^]^ and the simplified Tinetti test (Tinetti).^[^ ^35^, ^36^ ^]^

#### 2.1.3 Cognitive Outcomes

A comprehensive neuropsychological battery was administered by a neuropsychologist to assess different aspects of executive functioning, and included the following tests: the Trail making A & B test, the Stroop color‐word test, the Digit span forward and backward test, the WAIS‐III digit symbol‐coding test and the Frontal Assessment Battery (FAB).^[^ ^37^, ^38^, ^39^, ^40^, ^41^ ^]^ In addition, the Mini‐Mental State Examination (MMSE) and the Clock drawing tests were administered.^[^ ^42^, ^43^ ^]^ Both tests assess multiple cognitive domains including orientation, attention, memory, language, and visuospatial abilities for the MMSE, and visuospatial abilities and executive functioning for the Clock drawing test.

#### 2.1.4 Falls Outcomes

Falls were defined as “unintentionally coming to rest on ground, floor, or other lower level.”^[^ ^44^ ^]^ Prospective falls were monitored until the end of the 12‐month follow‐up or withdrawal from trial. They were recorded using a daily diary mailed monthly via prepaid preaddressed envelopes. If participants failed to return the diary, provided incomplete data, or reported falls, a blinded research assistant collected data on falls over the telephone or during face‐to‐face interview.

### 2.2 Adverse Events

Adverse events related to interventions were monitored continuously through adverse‐event case report forms and occurrence of side‐effects evaluated at each assessment visit occasion.

### 2.3 Statistical Analysis

Based on our primary outcome measure (i.e., stride variability under dual‐task condition) and data from a previous work, it was estimated that 96 subjects, (i.e., 48 per study arm) should be enrolled to obtain a statistical power of 90%, at a two‐sided level of significance of 5%, to detect a 1% difference between groups at 6 and 12 months, assuming that gait variability in this population is 4%. Assuming an attrition rate of 30% across the entire trial, a sample size of 138 subjects (i.e., 69 subjects per study arm) was thus targeted for the study.

Baseline characteristics were summarized by group using mean and standard deviation, or percentages. The “intent‐to‐treat (ITT)” approach was used as the primary analysis where participants were grouped according to their randomization assignment.

The estimates of within‐group differences and between‐group differences at 6 and 12 months were computed using the models described below.

Primary and secondary outcomes (i.e., gait, physical, and cognitive outcomes) were using linear mixed‐effects models, with random and fixed effects (intervention group assignment, time, and their interaction), without and with adjustment for age and sex. Random slopes and intercepts were included in the models. Gait variability measures were additionally adjusted for gait velocity.^[^ ^18^, ^45^ ^]^ The intervention groups were compared across time by testing the group‐by‐time interaction effects using these models. Effect sizes were calculated as Cohen's d for selected time points.

Regarding secondary outcomes of falls, relative risks comparing the number of participants with 1 or more falls and participants with multiple falls (≥2 falls) during the 12‐month trial period were estimated using log‐binomial regression models. The incidence rate ratio (IRR) for the number of falls was estimated using a negative binomial regression model. Survival analyses were also conducted: hazard ratios were estimated from a Cox proportional hazards model for the first fall, and the Andersen‐Gill model for all falls. Participants were censored at the end of follow‐up or at the time of the last follow‐up.

A two‐sided *P* value less than or equal to 0.05 was considered statistically significant. Statistical analyses were performed in Stata/IC (StataCorp) version 15.1.

## 3 Results

**Figure 1:**
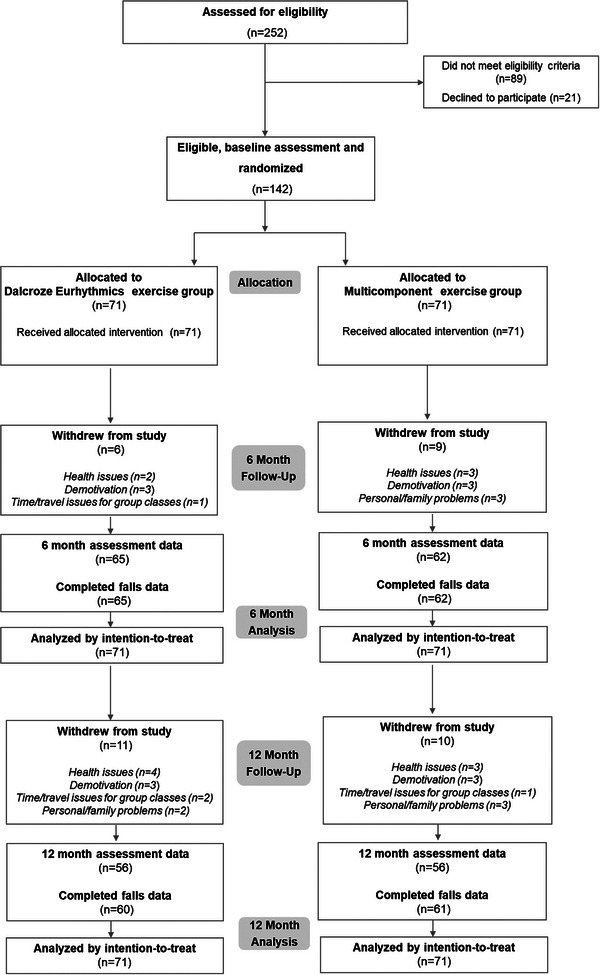
Flow of Participants in the EPHYCOS Randomized Clinical Trial.

**Figure** **1** depicts participant recruitment, enrollment, and follow‐up. Of 252 individuals screened, 142 fulfilled the eligibility criteria and were randomized (71 participants in each intervention group). Mean age of the participants was 74.3 ± 6.5 years and 92% were female (**Table** **1**). Across follow‐up, 15 (15/142, 11% in total) participants dropped out at 6 months and 6 additionally (21/142, 15% in total) at 12 months. There was no difference between intervention groups in the number of participants with incomplete follow‐up.

**Table 1:** Baseline Characteristics of Participants.

	Mean (SD)^a^ ^)^			
	MULTI group		DE group	
	(n = 71)		(n = 71)	
Age, y	73.8	(5.7)	74.8	(7.2)
Gender, No. (%)				
Male	8	(11)	4	(6)
Female	63	(89)	67	(94)
Live alone, No. (%)	38	(54)	41	(58)
Home help services, No. (%)	21	(30)	25	(35)
Height, cm	162	(9)	161	(8)
Body weight, kg	73	(17)	68	(13)
BMI, kg/m^2^	28	(5)	26	(5)
History of falls, No. (%)	62	(87)	60	(85)
Fall(s) in the past 12 months, No. (%)	34	(48)	35	(49)
Frailty criteria, Number of criteria^b^ ^)^	0.9	(0.8)	1.2	(0.9)
MNA short‐form, score	13.5	(0.9)	13.1	(1.5)
HADS anxiety subscale, score	6.4	(3.1)	5.3	(2.9)
HADS depression subscale, score	3.0	(3)	3.9	(2.4)
MMSE, score	27.3	(2.2)	27.2	(2.1)
Clock‐drawing test, score	9.3	(1.0)	8.9	(1.4)
Self‐rated health status^c^ ^)^	70.5	(18.6)	66.5	(19.6)
Self rated pain^c^ ^)^	25.4	(2.5)	28.0	(2.3)
Total number of medications	2.7	(2.0)	2.7	(2.0)
Short FES‐I, score	8.9	(2.3)	9.2	(2.3)

Abbreviations: SD, standard deviation; BMI, body mass index; MNA, Mini‐Nutritional Assessment; HADS, Hospital Anxiety and Depression Scale; MMSE, Mini‐Mental State Examination; FES‐I, Falls Efficacy Scale International.

^a)^ Unless otherwise indicated.

^b)^ According to Fried et al.^[^ ^46^ ^]^

^c)^ Scale from 0 = bad to 100 = excellent.

### 3.1 Adherence and Adverse Events

Across the 12‐month intervention period, on average, participants assigned to the DE intervention attended 81% of group‐based sessions (range 24% to 100%), while those assigned to the MULTI intervention attended 78% of group‐based sessions (range 35% to 100%). The mean attendance rates did not vary by intervention group. The mean attendance rate for the home‐based exercise sessions in the MULTI intervention group was 74% (range 28% to 100%). The main reasons given for not attending exercise classes were personal problems and travel issues. One serious adverse event related to the interventions was reported: one participant experienced an injurious fall (wrist fracture) during a MULTI group‐based exercise session.

### 3.2 Gait, Physical, and Cognitive Outcomes

**Figure 2:**
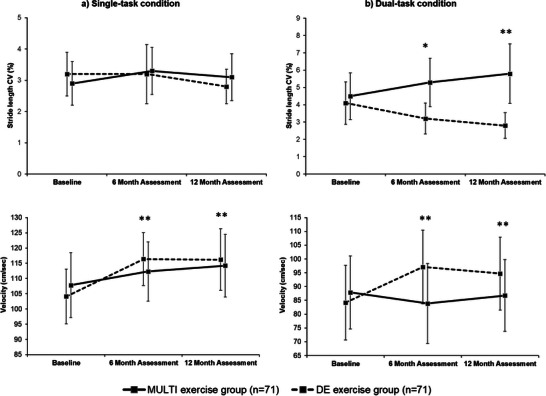
Stride length variability and gait velocity under a) single‐task condition and b) dual‐task condition for both groups. Values are means and standards deviations represented by vertical bars. CV: coefficient of variation. ^*^P < 0.05 ^**^P < 0.001.

**Table** **2** shows changes from baseline to 6 and 12 months in the two intervention groups for gait, physical and cognitive outcomes. At 12 months, participants in the DE intervention group had significant improvements in gait performances under dual‐task condition compared with MULTI group, including on the primary outcome of stride length CV (β from the adjusted model, −2.3; 95% CI, −3.1 to −1.4; p for interaction<0.001; d = −1.32), but also on gait velocity (**Figure** **2**) under both single‐ and dual‐task conditions (p < 0.05 for all).

**Table 2:** Changes in Gait, Physical and Cognitive Outcomes at 6 months and 12 months.

	Baseline (Mean, SD)		6 months (Mean, SD)		Interaction effect at 6 months (β, 95%CI)^a^	P−value^b^	Cohen's d effect size	12 months (Mean, SD)		Interaction effect at 12 months (β, 95%CI)^a^	P−value^b^	Cohen's d effect size
Outcomes	MULTI group (n=71)	DE group (n=71)	MULTI group	DE group				MULTI group	DE group			
**Gait**												
***Single−task condition***												
Gait velocity, cm/s	107.8 (21.2)	104.1 (17.9)	112.3 (19.2)	116.4 (17.4)	9.7 (5.1 to 14.3)	**<0.001**	0.82	114.2 (20.5)	116.2 (20.2)	8.9 (4.1 to 13.7)	**<0.001**	0.70
Cadence, steps/min	107.6 (12.4)	108.1 (11.1)	110.3 (10.7)	115.5 (8.2)	6.0 (3.3 to 8.8)	**<0.001**	0.90	111.6 (10.7)	115.6 (13.3)	5.5 (2.6 to 8.4)	**<0.001**	0.67
Stride length variability, %CV	2.9 (1.4)	3.2 (1.4)	3.3 (1.5)	3.2 (1.9)	−0.2 (−0.8 to 0.4)	0.512	−0.26	3.1 (1.5)	2.8 (1.1)	−0.4 (−1.0 to 0.2)	0.172	−0.54
***Dual−task condition***												
Gait velocity, cm/s	87.9 (26.4)	84.2 (27.1)	83.9 (29.1)	97.1 (26.7)	17.7 (12.7 to 22.7)	**<0.001**	1.24	86.8 (26.1)	94.7 (26.5)	16.7 (11.5 to 21.9)	**<0.001**	1.23
Cadence, steps/min	90.1 (17.9)	88.9 (22.0)	85.5 (20.7)	99.5 (17.4)	15.9 (11.4 to 20.4)	**<0.001**	1.26	88.4 (20.7)	98.8 (19.2)	14.8 (10.1 to 19.5)	**<0.001**	1.22
Stride length variability, %CV	4.5 (2.7)	4.1 (2.5)	5.3 (2.8)	3.2 (1.8)	−0.9 (−1.7 to −0.1)	**0.029**	−0.88	5.8 (3.4)	2.8 (1.5)	−2.3 (−3.1 to −1.4)	**<0.001**	−1.32
**Physical function**												
TUG, sec	10.8 (2.0)	11.3 (2.0)	10.4 (2.0)	10.3 (1.9)	−1.0 (−1.4 to −0.7)	**0.007**	−0.55	10.4 (2.4)	10.3 (1.9)	−1.2 (−1.5 to −0.8)	**0.003**	−0.47
SPPB, score	10.3 (1.5)	9.8 (1.7)	10.8 (1.3)	10.7 (1.3)	0.3 (−0.1 to 0.7)	0.100	0.28	10.8 (1.4)	10.8 (1.3)	0.3 (−0.1 to 0.7)	0.109	0.27
Standing balance, score^c^	3.7 (0.7)	3.5 (0.8)	3.7 (0.7)	3.8 (0.4)	0.3 (0.1 to 0.5)	**0.006**	0.47	3.8 (0.6)	3.9 (0.3)	0.3 (0.1 to 0.5)	**0.003**	0.44
Tinetti, score	0.7 (0.8)	0.9 (1.1)	0.6 (0.8)	0.5 (0.9)	−0.3 (−0.5 to −0.1)	**0.006**	−0.46	0.5 (0.8)	0.5 (0.9)	−0.3 (−0.5 to −0.1)	**0.011**	−0.40
Chair stand, sec^c^	12.3 (2.5)	12.9 (2.5)	11.5 (2.2)	12.4 (2.8)	0.3 (−0.5 to 1.0)	0.492	0.11	11.7 (2.2)	12.3 (3.2)	0.1 (−0.7 to 0.8)	0.866	0.03
**Cognitive function**												
MMSE, score	27.3 (2.4)	27.2 (2.1)	26.9 (2.1)	27.2 (2.0)	0.4 (−0.2 to 1.1)	0.207	0.27	27.0 (2.2)	27.2 (2.2)	0.4 (−0.3 to 1.1)	0.225	0.34
Clock−drawing test, score	9.3 (1.0)	8.9 (1.4)	8.9 (1.2)	9.3 (0.8)	0.7 (0.3 to 1.1)	**0.002**	0.57	8.8 (1.6)	8.8 (1.7)	0.3 (−0.1 to 0.8)	0.151	0.28
Trail making test Part A, sec	44.3 (13.4)	48.8 (15.5)	44.5 (16.2)	45.9 (17.6)	−2.9 (−8.0 to 2.3)	0.272	−0.18	43.3 (14.9)	45.2 (14.0)	−3.7 (−9.0 to 1.7)	0.180	−0.28
Trail making test Part B, sec	133.9 (74.3)	(67.3)	146.1 (0.2)	123.0 (52.7)	−16.8 (−31.9 to −1.6)	**0.030**	−0.32	138.6 (77.6)	120.8 (56.2)	−17.1 (−32.9 to −1.3)	**0.034**	−0.49
Digit span forward test, number of digits	5.8 (1.8)	6.1 (1.6)	5.8 (1.8)	6.0 (1.6)	−0.0 (−0.5 to 0.5)	0.974	0.09	6.2 (2.1)	6.2 (1.8)	−0.1 (−0.6 to 0.4)	0.685	−0.05
Digit span backward test, number of digits	4.9 (1.9)	4.4 (1.6)	4.7 (1.9)	5.1 (1.6)	0.9 (0.4 to 1.3)	**0.001**	0.61	5.0 (2.1)	5.1 (1.5)	0.6 (0.1 to 1.1)	**0.020**	0.50
WAIS−III digit symbol−coding tests, number	51.8 (12.5)	49.3 (11.4)	49.9 (13.3)	51.4 (14.0)	3.7 (1.2 to 6.1)	**0.004**	0.60	52.9 (13.2)	51.4 (13.8)	1.9 (−0.6 to 4.5)	0.140	0.29
FAB, score	15.9 (1.9)	16.1 (1.6)	15.5 (2.0)	15.9 (2.0)	(−0.5 to 0.7)	0.754	0.10	15.8 (1.8)	16.5 (1.5)	0.3 (−0.3 to 1.0)	0.316	0.32
Stroop color−word test / interference, sec	144.8 (43.2)	154.6 (41.1)	138.9 (42.1)	141.6 (36.4)	−5.3 (−18.1 to 7.6)	0.420	−0.16	141.7 (54.3)	140.0 (36.9)	−15.2 (−28.7 to −1.6)	**0.028**	−0.44

Regarding physical function, at 12 months, the DE intervention group had significant improvements in TUG test (−1.2; −1.5 to −0.8; p = 0.003; d = −0.47), Tinetti test (−0.3; −0.5 to −0.1; p = 0.011; d = −0.40), and standing balance test (0.3; 0.1 to 0.5; p = 0.003; d = 0.44), compared with MULTI group. No significant between‐group difference was observed on SPPB (p = 0.109) or chair stand test (p = 0.866), two tests on which both groups displayed significant improvements at 12 months (p < 0.05 for within‐group differences).

Regarding cognitive function, at 12 months, the DE intervention group also exhibited significant improvements compared with MULTI group in different executive‐functioning related tests, including on the Trail making test Part B (−17.1; −32.9 to −1.3; p = 0.034; d = −0.49), the Digit span backward test (0.6; 0.1 to 1.1; p = 0.020; d = 0.50), and the Stroop color‐word test (−15.2; −28.7 to −1.6; p = 0.028; d = −0.44). No significant between‐group difference was observed on other cognitive tests (p > 0.05).

### 3.3 Falls Outcomes

At 12 months, 95 falls were recorded (**Table** **3**). 37 falls were reported by 28/71 (39%) participants in the DE intervention group and 58 falls were reported by 36/71 (51%) participants in the MULTI intervention group. Four fractures occurred in each group (p > 0.05).

**Table 3:** Falls at the 12‐Month Follow‐up.

	MULTI Group	DE group			
Outcomes	(n = 71)	(n = 71)	Unadjusted^a^ ^)^		Adjusted^a^ ^),^ ^b^ ^)^
Falls (rate^c^ ^)^)	58 (0.8)	37 (0.5)			
Incidence Rate Ratio (95% CI)			**0.64 (0.41 to 0.98)** ^*^		**0.56 (0.36 to 0.87)** ^*^
Participants with ≥1 falls, No. (%)	36 (50.7)	28 (39.4)			
Relative Risk (95% CI)			0.78 (0.54 to 1.12)		0.72 (0.49 to 1.06)
Participants with multiple falls ≥2 falls, No. (%)	16 (22.5)	9 (12.7)			
Relative Risk (95% CI)			0.56 (0.27 to 1.19)		**0.42 (0.18 to 0.96)** ^*^
Survival analysis					
Hazard Ratio (95% CI) for first fall			0.60 (0.36 to 1.00)		**0.50 (0.29 to 0.87)** ^*^
Hazard Ratio (95% CI) for all falls			0.66 (0.43 to 1.00)		**0.58 (0.37 to 0.93)** ^*^

Abbreviations: CI, confidence interval.

^a)^ DE versus MULTI (reference).

^b)^ Adjusted for age, sex, history of falls over the previous 12 months, and Short Physical Performance Battery score.

^c)^ Fall rates per person/year.

* P<0.05

As compared with the MULTI group, the DE group experienced fewer falls during the intervention period: the unadjusted IRR for falls was 0.64 (95% CI, 0.41 to 0.98; p = 0.042). After adjustment for age, sex, history of falls over the previous 12 months, and SPPB score, the IRR for falls was 0.56 (95% CI, 0.36 to 0.87; p = 0.010). The number of participants with one or more falls was not statistically different between groups (unadjusted RR, 0.78; 95% CI, 0.54 to 1.12; p = 0.181), as was the number of participants with multiple falls (unadjusted RR, 0.56; 95% CI, 0.27 to 1.19; p = 0.131). Using a Cox proportional hazards model, the adjusted HR for the time to first fall was 0.50 (95% CI, 0.29 to 0.87; p = 0.014) in the DE group as compared with the MULTI group. Using the Andersen‐Gill model for all falls, the adjusted HR was 0.58 (95% CI, 0.37 to 0.93; p = 0.023) in the DE group as compared with the MULTI group.

## 4 Discussion

In the EPHYCOS comparative effectiveness trial, a DE exercise intervention was found to be more effective than a MULTI exercise intervention for improving a variety of physical and cognitive outcomes, and for falls reduction among community‐dwelling older adults at high risk for falls.

Our study showed that the DE program was effective in reducing the incidence of falls compared with the conventional evidence‐based multicomponent exercise program, with 42% fewer falls. Despite exercise is unanimously strongly promoted by current clinical practice guidelines, the scarcity of head‐to‐head comparative trials of proven evidence‐based exercise interventions remains a major critical issue. Such trials are of utmost importance to guide the clinicians’ decisions in exercise selection for their patients at high risk, given the numerous exercise regimens available for older adults. It is important to emphasize that the DE intervention demonstrated its superiority in terms of falls reduction despite administered at a lower intensity (one time weekly for the DE intervention versus two times weekly for the MULTI intervention), while the intervention groups had the same frequency of interactions with other study participants and the study staff.

Our results strengthen the evidence for physical‐cognitive interventions over more conventional exercise approaches.^[^ ^47^, ^48^, ^49^ ^]^ Especially, these results align with findings from a one of the few well‐designed comparative trials, showing a high magnitude of reduction in the incidence of falls with a Tai Ji Qan balance training intervention when compared to a conventional evidence‐based multicomponent exercise program.^[^ ^48^, ^49^ ^]^ Tai Ji Qan and DE interventions are both physical‐cognitive interventions that involve the coordination of different level of motor complexity and cognitive engagement, and stimulate simultaneously numerous physical and/or cognitive skills relevant to fall prevention. In the last decade, increased attention has been paid to the benefits of interventions combining motor and cognitive engagement (e.g., 3D Tai‐Ji or dance‐based activities using the ProFane taxonomy^[^ ^50^ ^]^), with an increased body of evidence highlighting the additional benefits resulting from such interventions for falls reduction, while the mechanism of the synergic effects remain poorly understood.^[^ ^47^, ^51^, ^52^, ^53^, ^54^, ^55^, ^56^, ^57^, ^58^ ^]^

The DE group had significant improvement in physical function as compared to the MULTI group, with especially benefits found on gait under dual‐task and balance performances, two strong risk factors for falls. Modest to large effect sizes were found, with the strongest effect observed into gait measures under dual‐task condition. These results especially converge with the growing data set supporting the added value of dual‐task training over single‐task for physical function.^[^ ^54^, ^59^, ^60^, ^61^ ^]^ Among the observed beneficial effects, DE exercise positively impacted gait under both single‐ and dual‐task conditions. Dual‐task/multi‐task paradigms are ecologically realistic proxies of situations that older adults are faced with during everyday life. The DE intervention reduced the primary outcome of gait variability under dual‐task, with a large effect size. Regarding the timing of progression, gait variability under dual‐task continuously improved over the 12 months, while overall greater improvements were achieved during the first 6 months on other physical and cognitive measures. Gait variability measures, especially under dual‐task, have been associated with fall risk and found to be more strongly associated with falls and mobility decline than routine spatio‐temporal measures.^[^ ^20^, ^21^, ^22^, ^23^, ^24^, ^25^ ^]^ Recently, a trial on Tai Ji Quan suggested that improvement in dual‐task ability may mediate improvement in physical and cognitive performances, as well falls observed with exercise.^[^ ^55^ ^]^ Several theories have been proposed in a recent systematic review to explain the physical improvements and reduction in falls following dual‐task training among older adults, the most prominent referring to a modulation of attention.^[^ ^47^ ^]^

Results for cognitive function, with significant improvements found in the DE group as compared to the MULTI group in several executive functioning‐related tests, with modest effect sizes, commensurate with our prior passive‐controlled study.^[^ ^17^ ^]^ DE exercise involves the performance of concurrent complex body movements/tasks while adapting/reacting to a range of environmental demands and stimuli (e.g., change of body movements with change in sound pitch of the piece or sequences in music).^[^ ^62^, ^63^ ^]^ The intervention appeals multiple cognitive skills, especially executive control skills, through exercises challenging the major core components of executive function: inhibition, cognitive flexibility, and working memory. This is likely to explain the improvements found on the Trail making test part B, the Digit span backward and the Stroop color‐word tests. Trial results are also in line with several systematic reviews and meta‐analyses documenting the benefits of physical‐cognitive interventions, especially under the form of dual‐task training, for executive function.^[^ ^26^, ^64^, ^65^, ^66^, ^67^, ^68^, ^69^ ^]^ It remains to be determined whether the addition of a specific cognitive/dual‐task training in the MULTI group lead to different results for cognitive outcomes.

Given the emerging data supporting close relationships between muscle health/physical function, cognition, and brain, improved cognitive and brain function might be an underlying mechanism behind the physical and falls benefits found with the DE intervention. Compelling evidence raises the possibility that interventions enhancing executive functions may translate to better physical performances (especially gait and balance performances), and in turn reduce fall risk.^[^ ^25^, ^26^, ^27^, ^28^, ^29^, ^30^, ^31^, ^70^, ^71^ ^]^ Thus, improved executive functioning, and associated functional plasticity, has the potential to be a strong mechanism by which DE intervention may improve muscle/physical performances and reduce falls. A further dedicated and adequately powered mechanistic trial should help to fully clarify whether DE exercise‐induced benefits in executive function or dual‐task ability serve as the mechanism linking to improved physical and falls outcomes. Regarding brain plasticity, in a recent study, our group showed a dysfunctional involvement of brain networks associated with motor control during a dual‐task performance (not during single‐task) in dynapenic older women, which was associated with worse performance in physical function tests.^[^ ^72^ ^]^ Thus, further research is needed to elucidate if DE‐induced benefits are mediated by neuronal effects.

Due to the multidimensional nature of the DE intervention (i.e., a multitask training, involving both motor and cognitive engagement, structured through rhythmic music) it remains unclear which component of the intervention was particularly beneficial. Especially, introducing rhythmic music stimulation has the potential to enhance the multitasking training benefits. There might be a strong interaction between musical rhythm perception, positive emotional experience, and neural activity within different structures of the motor and cognitive systems, which might play a key role in inducing motor and cognitive plasticity following DE training. Functional neuroimaging studies of music and rhythm processing have shown that, apart from auditory areas, music activates several structures associated with motor functions, including subcortical striatal nuclei cortical motor areas as well as the cerebellum. In addition, music increases activity in the limbic regions involved in emotion and reward, as well as higher‐order associative areas implicated in memory and other cognitive functions (e.g., hippocampus, prefrontal cortex). Importantly, listening to music has also been shown to produce an automatic―entrainment of various neural processes including motor, physiological, and even attentional systems.^[^ ^73^, ^74^, ^75^, ^76^ ^]^

Our study has important strengths, including i) the head‐to‐head comparative design of the trial, ii) the wide range of well‐validated measures that assessed several aspects of physical and cognitive function, and iii) the high retention and adherence rates. Notwithstanding these strengths, some limitations must be acknowledged. First, the results should be interpreted in the context of the eligibility criteria used in the EPHYCOS trial and with regard to the low representation of male participants. Also, as the interventions required participants to travel to attend group sessions in a community center, this may have resulted in the selection of older adults who were probably healthier and more socially‐connected, which would also impede generalizing findings to community‐dwelling older and frail adults as a whole. Second, due to the nature of the interventions, our trial was necessarily single‐blinded, but all participants were instructed not to divulge any aspect of their intervention to outcome assessors. Third, the multicomponent nature of the exercise interventions precluded to determine the relative contribution of each individual components to the beneficial effects. Fourth, there was no sham‐control group. Five, the intensity of interventions was not assessed (e.g., measures of heart rate and oxygen uptake), so we can't address any intensity‐related responses. Finally, the trial was conducted at a single site, thus a multicenter trial would be warranted to confirm the validity of the present study.

In conclusion, in older adults at high risk for falls, a DE exercise intervention was more effective than a MULTI exercise intervention to improve physical and cognitive function and reduce falls over 12 months. The mechanisms underlying DE exercise‐induced benefits remain to be fully elucidated.

## 5 Experimental Section

### Study Design

In a 12‐month randomized, single‐blind comparative effectiveness trial, participants at high risk for falls were randomized to receive either a DE exercise program (once weekly, group‐based) or a MULTI exercise program incorporating balance, functional, and strength training (twice weekly, mix of group‐ and home‐based), for 12 months (ClinicalTrials.gov Identifier: NCT01811745). The study protocol was approved by the State of Geneva's Ethics Committee (Protocol number 12–175). Written informed consent was obtained from all participants.

### Setting, Recruitment, and Participants

The trial was conducted in the Geneva area (Switzerland). The enrolment started in January 2013 and ended in January 2014. The last patient last visit was completed in February 2015. Participants were recruited in the community through several overlapping sources, including announcements in local media, public events, and targeted mailings.

Eligibility was established in a two‐step screening process. The first step was a screening telephone interview to assess specific inclusion and exclusion criteria. The second step was a screening visit including the administration of specific tests and questionnaires, and a physical examination by the study physician. Eligible participants were 1) adults 65 years or older, 2) community‐dweller, 3) identified as being at high risk for falls, and 4) willing to be randomly assigned to and adhere to a 12‐month exercise intervention. High risk for falls was defined by the presence of one of the following criteria: 1) one or more self‐reported falls after the age of 65 years, 2) balance impairment as assessed by a simplified Tinetti test with a score higher than 2 of 7,^[^ ^35^, ^36^ ^]^ or 3) one or two criteria of physical frailty.^[^ ^46^ ^]^ The following exclusion criteria were applied: 1) a medical history or physical examination revealing any major medical or physical condition that would preclude exercise or affect conduct of the trial (e.g., terminal illness), or 2) fully dependence on an assistive device, or 3) a diagnosis of dementia based on a comprehensive neuropsychological assessment, or 4) participation in a supervised DE exercise program or a supervised MULTI exercise program in the past 12 months.

### Randomization and Blinding

Eligible participants were randomly assigned in a 1:1 ratio to receive either the DE intervention or the MULTI intervention. Randomization occurred after baseline assessment according to a computer‐generated randomization sequence using a random permuted block design of randomly varying sizes between 2 and 6. Serially numbered, opaque, sealed envelopes containing the group assignment, opened consecutively in the presence of the participant, were used to ensure adequate concealment. Generation of the randomization sequence and preparation of envelopes was performed by an independent statistician.

All study assessors were blinded to group assignment and to the results of previous assessments. Participants were specifically instructed not to divulge any information to the assessors regarding their intervention allocation. The nature of the interventions precluded blinding of participants. Participants and exercise instructors were blind to the study's primary hypothesis. All statistical analyses were conducted by a blinded statistician.

### Interventions

Both interventions consisted of 12 months of supervised, structured, progressive, 1 h weekly exercise classes. In addition, participants randomized to the MULTI intervention were asked to undertake a half‐hour home exercise program, one time per week and during the entire period. All classes were conducted by qualified and experienced instructors trained to work with older people, in a group format of up to 15 participants. All instructors strictly followed a lesson plan to conduct their intervention.

Both intervention sessions were held in the same community facilities, including senior and community centers, located in a variety of places throughout the Geneva area.

Adherence to interventions was assessed via attendance lists completed by instructors (group‐based) and documentation on diaries completed by participants (home‐based).

*DE Exercise Intervention*: The evidence‐based DE exercise intervention program followed a protocol that was successfully used in a previous trial.^[^ ^18^ ^]^ Briefly, each class consisted of a warm‐up followed by varied multitask exercises and finished by a cooldown. Difficulty of exercises gradually increased over time. Core exercises consisted in walking following the improvised piano music and responding directly to its rhythmical changes. During the different courses of motion, the instructor could ask participants to simultaneously perform specific sequence of movements with the upper body, sometimes with the handling of objects (e.g., ball or percussion instruments), while interacting with the movement of other participants. Other typical exercises included quick task‐switching or inhibition exercises (e.g., walking out of rhythmic themes).

*MULTI Exercise Intervention*: The evidence‐based multicomponent exercise intervention program was tested in large scale trials. It includes core components for successful fall prevention in older adults, according to published guidelines.^[^ ^2^, ^5^, ^6^, ^8^, ^9^, ^10^, ^11^, ^12^, ^77^, ^78^, ^79^, ^80^ ^]^ Briefly, this multimodal exercise program contained progressive balance, functional, and strength training, with balance as a core component. Each class consisted of a warm‐up followed by static and dynamic activities including balance and gait tasks alternated with functional tasks and strength training activities. Particularly, exercises included: progressively difficult postures that gradually reduce the base of support and stress postural muscle groups (e.g., semi/tandem or heel/toes stance, one‐leg stance), dynamic movements that perturb the center of gravity (e.g., tandem/heel/toes walking, circle turns, reaching activities, forward/backward/sideways stepping and walking), tasks under reduced sensory input to challenge the sensory systems (e.g., stance with eyes closed), a specific strength training (e.g., knee extensor/flexor, hip abductors, ankle flexors/dorsiflexors), with a progressive increase in the amount of repetitions or weights lifted, and functional training (e.g., sit to stand, stepping over obstacles). The complexity of the tasks progressively increased over time. The home‐based exercise program was designed to be as similar to the supervised group‐based exercise program as possible. Participants received an intervention manual, including photographs and descriptions of exercises.

### Outcome Measures and Follow‐up

Participants were assessed at baseline and at 6‐month (midpoint) and 12‐month (intervention termination), following standardized protocols. Participants’ demographic and clinical characteristics were collected at enrolment through structured interview and a physical examination.

The primary outcome was the change in gait variability under dual‐task from baseline to 12 months. Secondary outcome measures were changes in other quantitative gait measures, physical tests performances, cognitive tests (especially executive tests) performances, and falls.

### Ethics Approval

The study was approved by the State of Geneva's Ethics Committee (Protocol number 12–175). All procedures performed were in accordance with the ethical standards of the institutional research committee and with the 1964 Helsinki declaration and its later amendments or comparable ethical standards.

## Conflict of Interest

The authors declare no conflict of interest.

## Author Contributions

A.T. had full access to all the data in the study and took responsibility for the integrity of the data and the accuracy of the data analysis. M.H., N.F., F.H., R.R., P.V., and A.T. contributed to the concept and design. M.H., N.F., F.H., R.R., S.F., C.G., P.V., and A.T. performed acquisition, analysis, or interpretation of data. M.H. and A.T. performed drafting of the manuscript. M.H., N.F., F.H., R.R., S.F., C.G., P.V., and A.T. performed critical revision of the manuscript for important intellectual content. M.H., F.H., and A.T. performed statistical analysis. R.R., P.V., and A.T. obtained funding for the study. F.H., R.R., S.F., C.G., P.V., and A.T. performed administrative, technical, or material support. A.T. performed supervision.

## Data Availability

The data that support the findings of this study are available on request from the corresponding author. The data are not publicly available due to privacy or ethical restrictions.
